# Retweet networks of the European Parliament: evaluation of the community structure

**DOI:** 10.1007/s41109-016-0001-4

**Published:** 2016-06-01

**Authors:** Darko Cherepnalkoski, Igor Mozetič

**Affiliations:** Jozef Stefan Institute, Jamova 39, SI-1000, Ljubljana, Slovenia

**Keywords:** Social networks, Networks of influence, Community detection, European Parliament

## Abstract

Analyzing information from social media to uncover underlying real-world phenomena is becoming widespread. The goal of this paper is to evaluate the role of Twitter in identifying communities of influence when the ‘ground truth’ is known. We consider the European Parliament (EP) Twitter users during a period of one year, in which they posted over 560,000 tweets. We represent the influence on Twitter by the number of retweets users get. We construct two networks of influence: (i) core, where both users are the EP members, and (ii) extended, where one user can be outside the EP. We compare the detected communities in both networks to the ‘ground truth’: the political group, country, and language of the EP members. The results show that the core network closely matches the political groups, while the extended network best reflects the country of origin. This provides empirical evidence that the formation of retweet networks and community detection are appropriate tools to reveal real-world relationships, and can be used to uncover hidden properties when the ‘ground truth’ is not known.

## Introduction

The ever-increasing social media and user-generated contents on the web is an abundant source of data which can provide relevant insight. This work is based on data from Twitter^1^, a social networking and micro blogging platform with over 300 million monthly active users, posting over 500 million tweets per day.

There are at least two approaches to analyzing the Twitter data: the (social) network analysis, and the contents analysis. In our previous research (Sluban et al. 2015), we combined both approaches. We detected influential communities, identified discussion topics, and determined the sentiment of the communities towards selected topics. However, the question whether the detected communities have corresponding real-world counterparts remained unanswered.

In this paper, we study retweet networks of the Members of the European Parliament and investigate their community structure. In particular, our goal is to determine what the community structure actually reflects. We approach this problem from the perspective of the network theory, which has been applied successfully to characterize a wide variety of complex systems. We show that the network theory is particularly effective at uncovering structure without prior knowledge of political orientation or national membership.

Twitter provides users with opportunities for different forms of interaction. The most prevalent form of interaction is following other users. When a user follows others, the tweets those users post are shown in the follower’s feed. A user can mention other users in a tweet, which brings the tweet to the attention of the users that are mentioned. Closely related to mentions are replies. A user can reply to a specific tweet from another user, engaging her/him in a direct conversation.

Retweets are the form of interaction most characteristic of Twitter as a social network. A user can retweet the tweets posted by other users. By doing this, the user creates another tweet with the exact same content as the original, with an additional attribution to the original tweet. This way, the information about the original author of the tweet is preserved. An idiosyncrasy of retweets is that the original tweet is always attributed in a retweet, eliminating the possibility of retweeting a retweet. When a user retweets a tweet, it is distributed to all her/his followers, just as if it were an originally authored tweet. Users retweet content that they find interesting or agreeable. Retweets have been analyzed in the context of information spreading and cascade formation. Additionally, retweets have been analyzed as a form of influence.

Existing research has analyzed various means of acquiring relevant tweets from the Twitter APIs (Kumar et al. 2015; Morstatter et al. 2013; Sampson et al. 2015) including the Streaming API used in this work, as well as improvements of the acquisition process by modification of queries.

To the best of our knowledge, there has been no previous work on the analysis of retweet networks of the Members of the European Parliament. Nevertheless, there is a considerable body of literature on several aspects relevant to this study.

Conover et al. 2011a predict the political alignment of Twitter users in the run-up to the 2010 US elections based on content and network structure. They analyze the polarization of retweet and mention networks for the same elections (Conover et al. 2011b). Borondo et al. 2012 analyze the user activity during the 2011 Spanish presidential elections. They additionally analyze the 2012 Catalan elections focusing on the interplay between the language and the community structure of the network (Borondo et al. 2014). Most existing research, as Larsson points out (Larsson 2014), focuses on the online behavior of political figures during election campaigns. Hix et al. 2009 investigate the voting cohesion of political groups in the European Parliament. Larsson 2014 examines the Twitter presence of representatives outside of election periods.

Lazer 2011 highlights the importance of the network science approaches in political science at large, since politics is a relational phenomenon at its core. Recent research has adopted the network approach to investigate the structure of legislative work in the US Congress, including committee and subcommittee membership (Porter et al. 2005), bill cosponsoring (Zhang et al. 2008), and roll-call votes (Waugh et al. 2009). In a more recent work, Dal Maso et al. 2014 examine the community structure with respect to political coalitions and government structure in the Italian Parliament.

As previously noted, there are three main modalities in which users on Twitter interact: 1) the user follows posts of other users, 2) the user responds to other user’s tweets by mentioning them or replying to them, and 3) the user forwards interesting tweets by retweeting them. Based on these three interaction types, one can define three measures of influence of a Twitter user: *indegree influence* (the number of followers, indicating the size of her/his audience), *mention influence* (the number of mentions of the user, indicating her/his ability to engage others in conversation), and *retweet influence* (the number of retweets, indicating the ability of the user to write content of interest to be forwarded to others).

Kwak et al. 2010 compare three different network-based measures of influence on Twitter: the number of followers, page-rank, and the number of retweets—finding the ranking of the most influential users differ depending on the measure. Cha et al. 2010 also compare three different measures of influence: the number of followers, the number of retweets, and the number of mentions—also finding that the most followed users do not necessarily score the highest on the other measures. Wang et al. 2010 compare the number of followers and page-rank with a modified page-rank measure that accounts for topic, again finding that ranking depends on the influence measure. Suh et al. 2010 investigate how different factors such as the account age, the use of hashtags and URLs impact the influence of the user measured by the number of retweets. Bakshy et al. 2011 investigate how information spreads on a retweet network and whether there are preconditions for the user to become influential. Boyd et al. 2010 examine retweets as a conversational practice and note that retweeting can be understood both as a form of information diffusion and as a means of participating in a diffuse conversation. Another perspective on Twitter analysis is to detect and focus on specific events instead of the complete time-frame (Kenett et al. 2014).

Along with the small-world phenomenon and power-law degree distribution, the most salient property that real-world networks exhibit is community structure, where network nodes are partitioned together in tightly knit groups, between which there are only loose connections (Girvan and Newman 2002). The identification of the community structure in the network is commonly based on the optimization of its modularity (Newman and Girvan 2004). Identification of communities in social networks is a very vibrant field of research. Many different algorithms exist which employ various approaches (Fortunato 2010). In this work, we perform community detection by the Louvain method, introduced by Blondel 2008, that is found to be among the best performing algorithms in a variaty of domains (Harenberg et al. 2014; Hric et al. 2014).

Evaluation of the community structure is often performed by qualitative comparison to the ground truth (Lužar et al. 2014; Perc 2010). The methodology for evaluating the degree to which the detected communities match known groups (Yang and Leskovec 2015), used in this work, is based on the *B* ^3^ algorithm (Bagga and Baldwin 2011; 2008). The *B* ^3^ measure is the most appropriate measure according to the formal constraints for extrinsic clustering evaluation measures proposed by Amigo 2009.

This paper is based on our preliminary work, presented in a workshop proceeding (Cherepnalkoski and Mozetič 2015), and extending it along three main dimensions. First, the collection process is extended from eight months to one year, increasing the data size by 50 %. Second, in addition to examining political group and country membership, we examine language as a potential factor along which the communities are formed. Finally, we investigate the indirect links between the countries in the retweet network in terms of tweet sharing between the EP members and the Europe-wide audience.

The paper is organized as follows. The section “The European Parliament on Twitter” describes the EU Parliament and the Twitter data collected. In the section “Community detection and evaluation”, we outline the Louvain community detection method and the measures to evaluate the detected communities w.r.t. the ‘ground truth’, i.e., the actual labels. The sections “Communities in the core network” and “Communities in the extended network” present the results of the community detection. The “Linking the EU countries in the extended network” section presents the results of the tweet sharing analysis between the countries. In “Conclusions”, we discuss the results and plans for future research.

## The European Parliament on Twitter

The European Union (EU) is a political and economic union which currently consists of 28 member states located in Europe. The EU operates through a system of supranational institutions which cover legislative, executive, judiciary, and monetary branches. The European Parliament, together with the Council of the European Union, is the principal legislative body.

### The European Parliament

The European Parliament (EP) functions analogously to national parliaments in traditional parliamentary democracies. It is elected every five years directly by the citizens of the EU countries. Member states are allocated a number of seats which roughly reflect the state’s population. The EP members are elected on a national basis, but sit in the EP according to political groups they belong to. They can address the EP in any of the 24 official languages of the EU, but data shows that they primarily use English.

Our work focuses on the period between October 1, 2014 and September 30, 2015. This period falls within the 8th EP which was elected on July 1, 2014. During this period, the EP consisted of 8 political groups:

1. European United Left–Nordic Green Left (*GUE-NGL*)—socialists and communists group,
2. Progressive Alliance of Socialists and Democrats (*S&D*)—social-democrats group,
3. The Greens-European Free Alliance (*Greens-EFA*)—greens and regionalists group,
4. Alliance of Liberals and Democrats for Europe (*ALDE*)—liberals group,
5. European People’s Party (*EPP*)—christian-democrats group,
6. European Conservatives and Reformists (*ECR*)—conservatives group,
7. Europe of Freedom and Direct Democracy (*EFDD*)—euroskeptics group, and
8. the Non-Attached Members (*NA*)—independents.

### Acquisition of tweets

We acquired the list of the EP members form the official site of the EP^2^. The list consists of 750 EP members. Their distribution according to political groups is presented in Table 1 (column *EP seats*). The official Twitter account of the EP, *Europarl_EN*, provides a list of Twitter accounts of the EP members^3^. We matched the EP members to the Twitter accounts and excluded Twitter accounts of former EP members; the result is a manually verified list of 546 Twitter accounts of all the EP members which have one. The distribution of the EP members with Twitter accounts according to political groups is given in Table 1 (column *Twitter accounts*).

**Table 1:** The number of Twitter users in the European Parliament by political group

Group	EP seats	Twitter accounts	Core network	Extended network	
GUE-NGL	52	36	35	35	
S &D	191	151	127	136	
Greens-EFA	50	50	43	45	
ALDE	68	50	45	49	
EPP	218	152	121	139	
ECR	72	49	38	44	
EFDD	47	35	27	31	
NA	52	28	23	28	
Total	750	546	459	507	

We monitored the activity related to the official accounts of the EP members through the Twitter Streaming API^4^. For each member, we acquired all their tweets as well as all the replies and retweets.

**Fig. 1:**
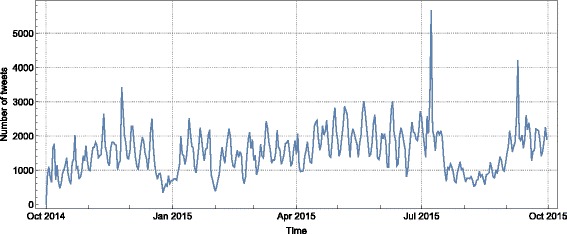
The total number of tweets posted by all the EP members per day. The number of tweets ranges between 500 during low-activity periods, such as Christmas holidays and summer months, and can reach over 5,000 in high-activity periods

Within the period of our analysis—between October 1, 2014 and September 30, 2015—the EP members have posted 561,255 tweets, of which 295,395 (53 %) are originally authored and the other 265,860 (47 %) are retweets. On average, all EP members together posted 1,538 tweets per day, and each active member posted on average 3.1 tweets per day (Fig. 1).

Together with each tweet, Twitter provides metadata about the tweet which includes the identified language of the tweet. We use this information to determine the language an EP member uses on Twitter. For each EP member, we compute the distribution of languages across all of the tweets posted by her/him. We determine the most commonly used language and use this language in the analysis. In some cases, Twitter is unable to determine the language of a tweet and reports the language as ‘undetermined’. If most of the tweets of an EP member are categorized as ‘undetermined’, we consider him/her as using ‘undetermined’ as their preferred language.

### Construction of retweet networks

The collected tweets described in the previous section are used to construct retweet networks. A retweet network is a directed weighted graph, where nodes represent Twitter users and edges represent the retweet relation. The direction of an edge corresponds to the direction of information spreading or influence; the weight of the edge is the number of times one user retweets the other. We construct two retweet networks: (i) the core network, containing as nodes only the EP members and (ii) the extended network, containing as nodes the EP members and all other users which have retweeted or have been retweeted by an EP member.

The core network consists of 459 nodes and 4,441 edges. The distribution of nodes according to political groups is in Table 1 (column *Core network*). The extended network consists of 498,103 nodes, of which 507 are the EP members, and 808,505 edges. The distribution of the member nodes according to political groups is also in Table 1 (column *Extended network*). Note that there are more EP members in the extended network (507) than in the core network (459) since 48 EP members (the difference) have been retweeted only by non-EP members. The extended network is much larger, and at the same time much sparser than the core network. The sparsity of the extended network is expected. While the core network represents interactions between the EP members, the interactions between the EP members in the extended network are dwarfed by the interactions between the general public on Twitter and the EP members. These interactions are mostly unidirectional, represented as retweets by the general public of the tweets posted by the EP members. An overview of the size and modularity of both networks is in Table 2.

**Table 2:** The size of the two retweet networks

	Cor enetwork	Extended network
Nodes	459	498,103
Edges	4,441	808,505
Retweets	17,062	3,683,967
Detected		
communities	8	16
Modularity	0.506	0.762

## Community detection and evaluation

We want to determine which characteristics of the EP members are reflected in the detected communities in the network. We investigate three possible factors that influence the formation of communities in the retweet networks.

- The political group whose members they are. In a national parliament, the political party is the most determining characteristic of a member of the parliament. In the EP, however, the political groups do not provide neither funding nor any support during the election process.
- The country they come from. Each member of the EP is elected in a member state of the EU. The seats in the EP are allocated on a per country bases, and the members represent that country in the parliament.
- The language they tweet in. The language the EP members use reflects the audience they address. Members which come from countries which use the same language have more opportunities to share their messages.

### Community detection

**Fig. 2:**
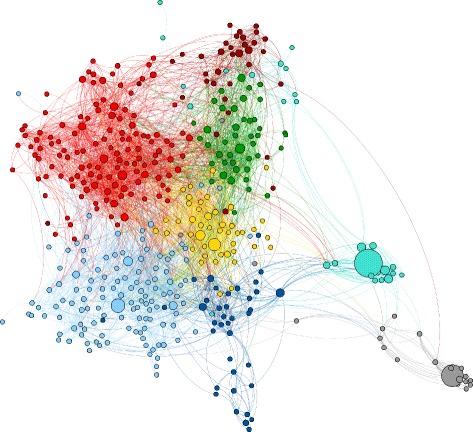
The core retweet network of the EP members. The nodes represent the EP members, and the links represent the retweet relations. The node size is proportional to the total number of times the member has been retweeted by other members. The node colors correspond to the political groups of the EP members and the link colors to the EP member that has been retweeted. There is a clear segregation of the EP members from different groups

**Fig. 3:**
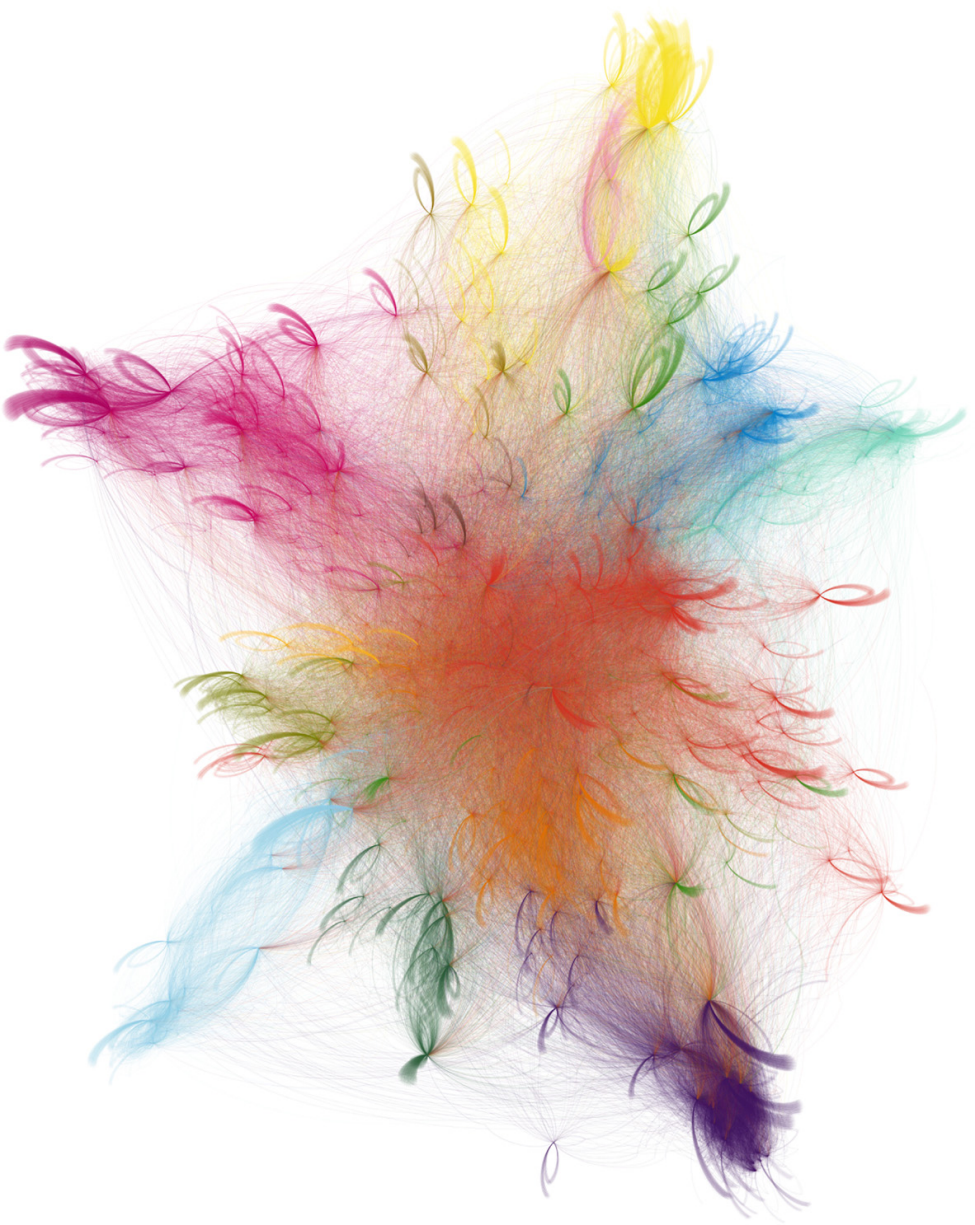
The extended retweet network of the EP members. The nodes represent the EP members and Twitter users which have retweeted or been retweeted by them. The links represent the retweet relations. The colors correspond to the detected communities

In Fig. 2, we present the core network with a force-directed layout, where the color of the node identifies the political group of the EP member. There is an intuitive visual grouping of the EP members according to political groups. In Fig. 3, the extended network with the detected communities is presented.

The goal of most community detection algorithms, implicit or explicit, is to find the best trade-off between a large intra-cluster density and a small inter-cluster density. Community detection algorithms perform maximization of modularity (Newman 2006). A good partitioning of a network in communities is one in which there are fewer than expected edges between the communities. The modularity is, up to a multiplicative constant, the number of edges falling within groups minus the expected number in an equivalent network with edges placed at random. Previous work on roll-call votes suggests that the result of modularity optimization should find groups and coalitions in a parliament (Dal Maso et al. 2014).

We perform community detection using the well established Louvain algorithm (Blondel et al. 2008). The Louvain method is a computationally very efficient algorithm that is well suited for large networks. It optimizes modularity through an iterative heuristic approach that consists of two repeating phases. In the first phase, modularity is optimized by allowing only local changes in communities; in the second, a new network is build that consists of one node for each previously found community. The algorithm repeats the iterations until the first phase can make no further improvements in modularity.

### Evaluation measures

To asses how closely the detected communities correspond to actual groups, we use the *B* ^3^ measure (Bagga and Baldwin 2008), which is considered the most appropriate measure for extrinsic evaluation of clustering (Amigó et al. 2009). The *B* ^3^ measure decomposes the evaluation into calculating the precision and recall associated with each node in the network. Let *N* be the set of all nodes in the network. For each node *n*∈*N*, we denote as *L*(*n*) the set of nodes which have the same label as *n*, i.e., members of the same actual group. With *C*(*n*), we denote the set of all nodes which are members of the same community as *n*. The *B* ^3^ precision of a node *n*, *P*(*n*), is computed as the fraction of nodes which have the same label and are in the same community as *n*, from all the nodes which are in the same community as *n*. Similarly, the recall of a node *n*, *R*(*n*), is computed as the fraction of nodes with the same label and in the same community, from all the nodes with the same label as *n*.

1 $$\begin{array}{*{20}l} P(n) &= \frac{\left|L(n)\cap C(n)\right|}{\left|C(n)\right|} \end{array} $$

2 $$\begin{array}{*{20}l} R(n) &= \frac{\left|L(n)\cap C(n)\right|}{\left|L(n)\right|} \end{array} $$

The precision and recall can be further combined into an *F*_1_ score, which is a harmonic mean of the precision and recall:

3 $$ F_{1}(n) = 2\,\frac{P(n)\,R(n)}{P(n) + R(n)}  $$

The *F*_1_ score is a special case of Van Rijsbergen’s effectiveness measure (Van Rijsbergen 1979), where precision and recall can be combined with different weights.

The precision reflects the homogeneity of the community. The lower the number of actual groups in the community, the higher the precision. Conversely, the recall reflects to compactness of the actual group. The lower the number of detected communities in an actual group, the higher the recall. The *F*_1_ score balances the precision and recall.

Furthermore, to quantify how well an actual group is reflected in the community structure of the network, we calculate the mean precision, recall, and *F*_1_ of the EP members for each group. Let {*L*_1_,*L*_2_,…,*L*_*k*_} be the partitioning of the nodes according to actual labels. The precision, recall, and *F*_1_ score of the set of the nodes *L*_*i*_ are computed as:

4 $$\begin{array}{*{20}l} P(L_{i}) &= \frac{1}{\left|L_{i}\right|}\sum_{n\in L_{i}}P(n) \end{array} $$

5 $$\begin{array}{*{20}l} R(L_{i}) &= \frac{1}{\left|L_{i}\right|}\sum_{n\in L_{i}}R(n) \end{array} $$

6 $$\begin{array}{*{20}l} F_{1}(L_{i}) &= \frac{1}{\left|L_{i}\right|}\sum_{n\in L_{i}}F_{1}(n) \end{array} $$

## Communities in the core network

**Fig. 4:**
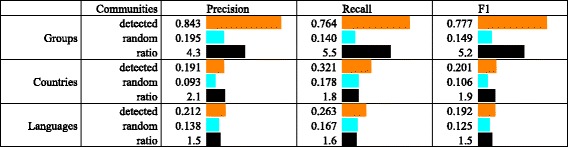
The core network: a comparison of the 8 detected and random communities. The comparison is with respect to the political groups, countries, and languages. The results show that the community structure best reflects the partitioning in political groups

Community detection in the core network results in a partitioning into 8 communities with a modularity score of 0.506. We evaluate how closely the partitioning in communities corresponds to the partitioning according to the three factors we investigate. The results are summarized in Fig. 4. In the following subsections, we present the results in detail.

### Communities and political groups

**Fig. 5:**
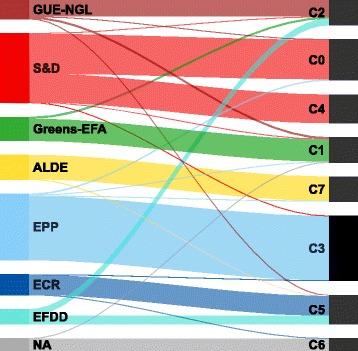
Distribution of the political groups across the 8 detected communities in the core network. The size of the bands between the political groups and communities is proportional to the number of the EP members. The results show that all political groups, except *S&D* and *EFDD*, are contained predominantly in single communities

Figure 5 shows the correspondence between the political groups and detected communities. First, it points out how members of different political groups are distributed across communities. Generally, most of the members of one group are located in a single community. The EP members from *S&D*, however, are divided almost evenly into two communities (C0 and C4); the members from *EFDD*, are also divided into two communities (C2 and C5). Second, Fig. 5 also shows the composition of communities with respect to political groups. In general, the communities consist mostly of members of a single group. Notable exceptions are community C2 which contains many EP members from both *GUE-NGL* and *EFDD*, and community C5 which contains EP members from *ECR* and *EFDD*.

We calculate the mean precision, recall, and *F*_1_ score for the core network to characterize how well the community structure reflects the political groups of the EP members. The results are shown in Fig. 4 (rows *Groups*). The precision is high, 0.843, which reflects the fact that most of the communities, with the exception of C2 and C5, are dominated by a single political group. The recall is moderately high, 0.764, which reflects the fact that most of the political groups, with the exception of *S&D* and *EFDD*, are predominantly contained within a single community. The *F*_1_ score is also moderately high, 0.777. In comparison, a random partitioning of the graph into 8 communities has (on average over 1000 random partitionings) precision of 0.195, which is over 4 times lower, recall of 0.140, which is over 5 times lower, and *F*_1_ score of 0.149, which is also over 5 times lower than the scores obtained with the partitioning into communities.

**Fig. 6:**
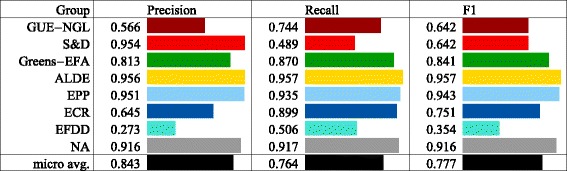
The core network: correspondence measures between the political groups and the 8 detected communities. The results show that most of the groups, with the exception of *EFDD*, are well reflected in the community structure

The correspondence measures for the political groups are presented in Fig. 6. *GUE-NGL* has an average precision (0.566) and moderately high recall (0.744), which corresponds to its members being dispersed in several groups, in only one of which they constitute the majority. *S&D* has a very high precision (0.954) and the lowest recall (0.489), as a result of being almost perfectly split into two communities where its members are an overwhelming majority. *Greens-EFA* has a high precision (0.813) and recall (0.870) because its members are mostly contained in a single community where they are a majority. *ALDE* has the highest precision (0.956) and recall (0.957) due to the fact that almost all of its members are contained in a single community which contains very few others. *EPP* has a very high precision (0.951) and recall (0.935) since it members are predominantly contained in one large community with only a few other members. *ECR* has above average precision (0.645) and high recall (0.899) as a consequence of being contained predominantly in a single community which contains almost as many members from other groups. *EFDD* has the lowest precision (0.273) and an average recall (0.506) resulting from the fact that it is evenly divided into two communities, in none of which its members are a majority. And finally, *NA* has very high precision (0.916) and recall (0.917) corresponding to the largest part of its members being in a single community which contains only one member from another political group.

### Communities and countries

We also evaluate the correspondence between the EU countries and the detected communities. We again calculate the mean precision, recall, and *F*_1_ score for the network to characterize how well the community structure reflects the country membership of the EP members. The results are shown in Fig. 4 (rows *Countries*). Both precision and recall are low, 0.191 and 0.321 respectively, which shows that communities in the core network and not organized along country membership. In comparison, a random partitioning of the graph into 8 partitions has (on average over 1000 random partitionings) precision of 0.093, recall of 0.178, and *F*_1_ score of 0.106, which are all only around 2 times lower than the scores obtained with the partitioning into communities. We do not further investigate the differences between individual countries since the overall correspondence between the countries and the communities is low.

### Communities and languages

Lastly, we evaluate the correspondence between languages and communities. The results are shown in Fig. 4 (rows *Languages*). Again, precision and recall are both low, 0.212 and 0.263 respectively, which shows that communities in the core network and not organized along the language in which EP members post tweets. In comparison, a random partitioning of the graph into 8 partitions has (on average over 1000 random partitionings) precision of 0.138, recall of 0.167, and *F*_1_ score of 0.125, which are all only 1.5 times lower than the scores obtained with the partitioning into communities. Again, we do not further investigate the differences between individual languages since the overall correspondence between the languages and the communities is low.

## Communities in the extended network

The extended network consists of the EP members as well as all other users which have retweeted or have been retweeted by the EP members. As such, it is several orders of magnitude larger than the core network. Moreover, the edges from non-EP members to the members far outnumber the edges between the EP members. This network reflects the retweeting practice of the general public when it comes to political issues. In this case, we again investigate three alternatives: Is the partitioning of the network in communities dominated by the political groups, by the countries of origin of the EP members, or by the language in which they post their tweets?

**Fig. 7:**
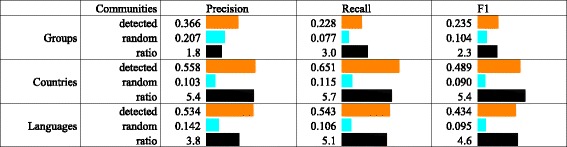
The extended network: a comparison of the 16 detected and random communities, with respect to the political groups, countries, and languages. The results show that the community structure best reflects the partitioning according to the country of origin

We again apply the Louvain method for community detection which results in 16 communities with a modularity score of 0.762. The high modularity score stems from the fact that many Twitter users retweet only a single or a few EP members. A force-directed layout of the network, colored by the detected communities is presented in Fig. 3. For further analysis, we focus only on the EP members—for them, we know the ‘ground truth’, i.e., the political group, the country which they represent, and the language they use on Twitter. The results are summarized in Fig. 7.

### Communities and political groups

Analogously to the core network, we analyze how closely the partitioning in communities corresponds to the partitioning in political groups.

The mean precision, recall, and *F*_1_ score for the extended network, which characterize how well the community structure reflects the political group membership, are presented in Fig. 7 (rows *Groups*). Both precision and recall (and subsequently *F*_1_) are low. In comparison, a random partitioning of the graph into 16 partitions has (on average over 1000 random partitionings) precision which is almost 2 times lower, recall which is 3 times lower, and *F*_1_ score which is around 2 times lower than the ones obtained with the partitioning into communities. These values are still substantially lower than the ones obtained for the core network with respect to political groups.

### Communities and countries

**Fig. 8:**
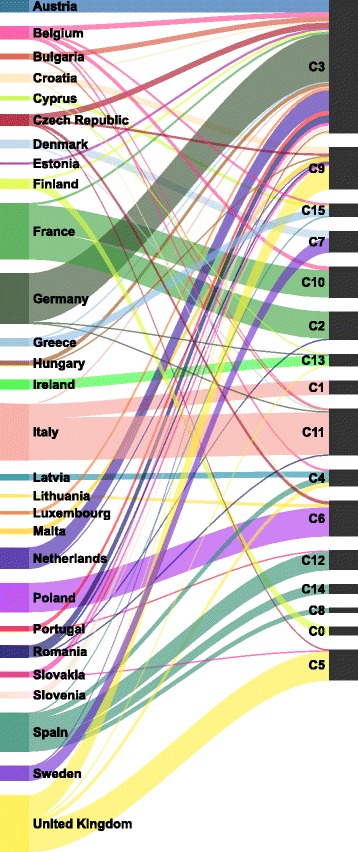
Distribution of the EU countries across the 16 detected communities in the extended network. The size of the bands between the countries and communities is proportional to the number of EP members. The results show that members from most of the countries are contained predominantly in single communities. Notable exceptions are the members from France, Spain, and United Kingdom

We next investigate how the country of origin of the EP members is reflected in the community structure. To this end, we evaluate the matching between the 16 communities and the 28 EU countries. Figure 8 illustrates how members from different EU countries are spread across the detected communities. Most countries have their members contained within only a few communities. Moreover, for the majority of countries, one community contains the prevailing number of the its members.

The evaluation results for the partitioning in countries are presented in Fig. 7 (rows *Countries*). In comparison to the partitioning in political groups, they are substantially higher. We also evaluated the average random partitioning, which has precision, recall, and *F*_1_ score that are around 5.5 times lower than the ones obtained with the partitioning into communities. These values are comparable with those for the partitioning in political groups of the core network.

**Fig. 9:**
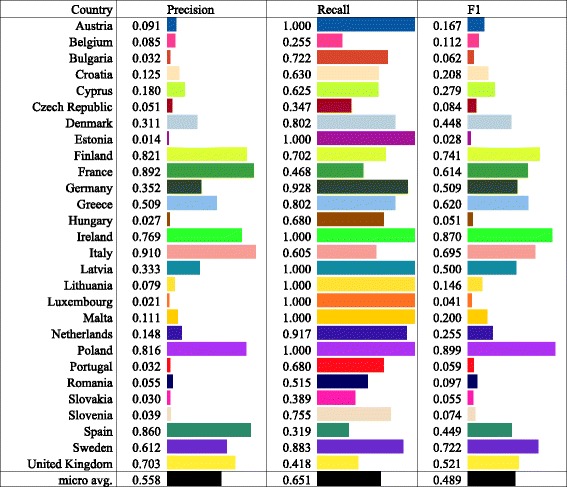
The extended network: correspondence measures between the EU countries and the 16 detected communities. The results show that about half of the countries, mostly larger, are well reflected in the detected communities

Figure 9 shows the mean precision, recall, and *F*_1_ score for each EU country. The *F*_1_ scores for the different countries vary substantially, ranging from 0.028 for Estonia to 0.899 for Poland.

### Communities and languages

**Fig. 10:**
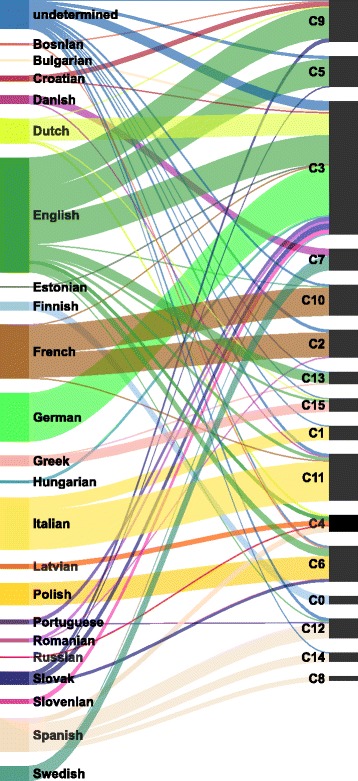
Distribution of languages used on Twitter across the 16 detected communities in the extended network. The size of the bands between the languages and communities is proportional to the number of the EP members. The results show that many of the languages, most notably English and Spanish, are dispersed to several communities. In addition, the ’undetermined’ category is also dispersed to virtually all the communities

Finally, we investigate how the language of Twitter posts of the EP members is reflected in the community structure. Hence, we evaluate the correspondence between the 16 detected communities and the 23 languages used on Twitter. We rely on the Twitter language recognition which is not perfect. Figure 10 illustrates how different languages used by the EP members are spread across the detected communities. The size of each language group in Fig. 10 is proportional to the number of the EP members who primarily use that language. Note that the community identifiers are the same as in Fig. 8.

The evaluation measures for the partitioning by language are presented in Fig. 7 (rows *Languages*). In comparison to the partitioning by countries, they are slightly lower. This is an interesting result since one would expect that common language used on Twitter is more important than the country of origin.

## Linking the EU countries in the extended network

The results in the previous section indicate that the communities in the extended network best reflect the country of origin of the EP members. Figure 8 shows a bipartite network of the EU countries and the detected communities. We project the bipartite network to a unipartite network of countries by defining an appropriate weighting of the network edges. The country network thus represents links between the EU countries in terms of the shared tweets between the EP members and the Europe-wide audience. The sharing of tweets (retweeting) is captured by the detected communities in the extended retweet network.

The weighting of the country network edges is based on the following intuition. A weight should be large when many EP members from a pair of countries occur within only a few communities, and small when only a few members from the two countries share a community. In the extreme, the weight should be 0 when the EP members from the two countries are not together in any community, and 1 when all the members from both countries occur together in the same community.

A weight of the edge between two countries is computed from the number of pairs of the EP members. Let *A* and *B* be two countries and let the index set {1,2,…,*N*} denote the communities. Let *A*_*C*_=(*A*_1_,*A*_2_,…,*A*_*N*_) and *B*_*C*_=(*B*_1_,*B*_2_,…,*B*_*N*_) be the vectors of assignment of the EP members from *A* and *B* to the communities. We count the number of pairs of members, where one is from *A* and the other from *B*, within the same community. The number of such pairs is given by the dot product *A*_*C*_·*B*_*C*_. We define the weight *w*(*A*,*B*) as the fraction of all the pairs which are in the same community:

7 $$ w(A,B) = \frac{A_{C} \cdot B_{C}}{|A|\,|B|}  $$

The resulting weight is a real number in the interval [0,1].

**Fig. 11:**
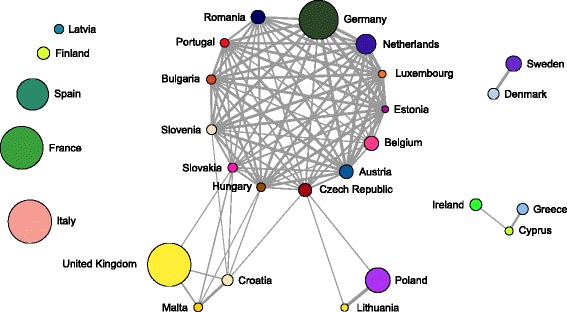
A network of the EU countries, linked by tweet sharing, is formed from the bipartite network in Fig. 8. The weight threshold is 0.2. The size of a node is proportional to the number of the EP members from the country. The network contains one large connected component consisting of 18 countries, with a core of 13 countries that are densely connected

We visualize the tweet sharing links between the countries as a weighted network. Figure 11 shows the country network with all the weights ≥0.2. The network contains one large connected component consisting of 18 countries. Within the large connected component, there is a core of 13 countries which are densely connected, and two tentacles of two (Poland and Lithuania) and three countries (United Kingdom, Malta, and Croatia). In addition, there are two smaller connected components with two countries (Sweden and Denmark), and three countries (Greece, Cyprus, and Ireland). The most interesting are the five countries, disconnected from the rest (Italy, France, Spain, Finland, and Latvia) since they apparently do not participate in tweet sharing.

**Fig. 12:**
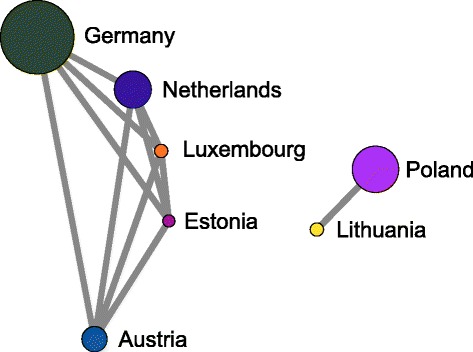
Connected components of the country network from Fig. 11 with the weight threshold of 0.9. At this high threshold, only two major components persist. The larger one consist of five countries (Germany, Netherlands, Austria, Luxembourg, and Estonia) and forms the core of the largest connected component in Fig. 11

Figure 12 shows the connected components of the country network with all the weights ≥0.9. At this high threshold, only two major components persist. One consists of five countries (Germany, Netherlands, Austria, Luxembourg, and Estonia) which forms the core of the largest connected component in Fig. 11. The other, consisting of Poland and Lithuania, is a tentacle of the largest connected component in Fig. 11.

## Conclusions

In this paper we investigate the retweeting behavior of the EP members in a period of one year. We use Twitter data to identify communities of influence and evaluate the detected communities with respect to the known ‘ground truth’. The analysis reproduces the actual political groups and countries of origin of the EP members, without prior assumptions. A summary of the *F*_1_ scores for the core and the extended networks, in comparison to the political groups, countries, and languages for the detected and random communities, is given in Table 3.

**Table 3:** A summary of the *F*_1_ scores

	Communities	**Groups** (8)	**Countries** (28)	**Languages** (23)
**Core**	detected (8)	**0.777**	0.201	0.192
**network**	random (8)	0.149	0.106	0.125
**Extended**	detected (16)	0.235	**0.489**	0.434
**network**	random (16)	0.104	0.090	0.095

Highlighted are the highest scores for each network

The results suggest that the retweeting behavior of the EP members is driven by their political group membership. On the other hand, the retweeting behavior of the Twitter audience which follows the activities of the EP members is driven by their country of origin. Surprisingly, the language of the EP members used on Twitter does not dominate the retweeting behavior of neither the EP members, nor the general public.

Existing research (Hric et al. 2014) points out that most real world networks do not have clear ‘ground-truth’ counterparts to the detected communities regardless of which community detection algorithm is used. In this work, we show that the communities in the two retweet networks of MEPs have very clear counterparts, namely, political groups and countries.

We have already successfully applied the Louvain method for community detection to uncover influential communities in retweet networks, albeit in the context of climate and energy issues (Sluban et al. 2015). The results of the present study reinforce the suitability of the Louvain method for uncovering communities in retweet networks. In our preliminary work (Cherepnalkoski and Mozetič 2015), we have also performed community detection by hierarchical stochastic block modeling (Peixoto 2014). The first experiments, however, resulted in substantially larger numbers of detected communities and in considerably lower *F*_1_ scores.

Community detection is a very vibrant field of research. There are multiple studies focused on comparison of algorithms for community detection (Fortunato 2010; Harenberg et al. 2014; Hric et al. 2014). Even tough comparing different community detection algorithms is important on its own, we plan to focus our future research in the following three key areas.

The presence and activities of the EP members on Twitter can be coupled with their actions in the Parliament. We plan to investigate the relations between the retweet networks and the roll-call vote networks. One of the findings of this study is that community detection can recreate the structure of different political groups with different degrees of effectiveness. Different political groups, also, manifest different levels of coherency in their voting behavior. Investigating whether these two phenomena are related will contribute to the overarching theme of engagement in social media by elected representatives.

So far, we have disregarded the contents of the tweets posted, and focused on the aggregated retweet behavior only. The spreading of influence on Twitter is, however, dependant on the discussion topics. Different topics are accompanied by different levels of agreement and controversy, and may bring two political groups closer together or move them further apart. We plan to implement topic detection on Twitter data, and investigate how different topics influence the community structure of the retweet network of the EP members.

Different topics convey different sentiment. Sentiment analysis can be applied to uncover the attitude of different communities toward various issues. We have already applied the sentiment analysis to various domains, such as: (i) to compare the sentiment leaning of different network communities towards various environmental topics (Sluban et al. 2015), (ii) to study the emotional dynamics of Facebook comments on conspiracy theories (Zollo et al. 2015), (iii) to analyze the effects of Twitter sentiment on stock prices (Ranco et al. 2015), (iv) to monitor the sentiment about political parties before and after the elections (Smailović et al. 2015), and (v) to rank the widely used emojis by sentiment (Kralj Novak et al. 2015). In the future we plan to employ sentiment analysis to characterize the sentiment of the EP political groups towards different policy and regulation issues.

## Endnotes

^1^ http://www.twitter.com/

^2^ http://www.europarl.europa.eu/meps/en/full-list.html (accessed June 1, 2015)

^3^ https://twitter.com/Europarl_EN/lists/all-meps-on-twitter/members (accessed September 30, 2014)

^4^ https://dev.twitter.com/streaming/overview/request-parameters

## References

[CR1] Amigó E, Gonzalo J, Artiles J, Verdejo F (2009). A comparison of extrinsic clustering evaluation metrics based on formal constraints. Inf Retrieval.

[CR2] Bagga, A, Baldwin B (2011) Algorithms for scoring coreference chains In: Proc. 1st Intl. Conf. on Language Resources and Evaluation Workshop on Linguistics Coreference, 563–566.

[CR3] Bagga A, Baldwin B (2008). Entity-based cross-document coreferencing using the vector space model. Proc. 17th Intl. Conf. on Computational Linguistics (COLING).

[CR4] Bakshy E, Hofman JM, Mason WA, Watts DJ (2011). Everyone’s an influencer: Quantifying influence on twitter. Proc. 4th ACM Intl. Conf. on Web Search and Data Mining (WSDM).

[CR5] Borondo J, Morales AJ, Losada JC, Benito RM (2012). Characterizing and modeling an electoral campaign in the context of Twitter: 2011 Spanish Presidential election as a case study. Chaos.

[CR6] Borondo J, Morales AJ, Benito RM, Losada JC (2014). Mapping the online communication patterns of political conversations. Physica A: Stat Mech Appl.

[CR7] Boyd, D, Golder S, Lotan G (2010) Tweet, tweet, retweet: Conversational aspects of retweeting on twitter In: Proc. 43rd Hawaii Intl. Conf. on System Sciences (HICSS), 1–10.

[CR8] Blondel VD, Guillaume JL, Lambiotte R, Lefebvre E (2008). Fast unfolding of communities in large networks. J Stat Mech Theory Exp.

[CR9] Cha, M, Haddadi H, Benevenuto F, Gummadi PK (2010) Measuring user influence in twitter: The million follower fallacy In: Proc. Intl. AAAI Conf. on Weblogs and Social Media (ICWSM), 10–17.

[CR10] Cherepnalkoski, D, Mozetič I (2015) A retweet network analysis of the European Parliament In: Proc. 11th Intl. Conf. on Signal-Image Technology & Internet-Based Systems (SITIS), 350–357, doi:10.1109/SITIS.2015.8.

[CR11] Conover, M, Gonçalves B, Ratkiewicz J, Flammini A, Menczer F (2011a) Predicting the political alignment of twitter users In: Proc. 3rd IEEE Conf. on Social Computing (SocialCom).

[CR12] Conover, M, Ratkiewicz J, Francisco M, Gonçalves B, Flammini A, Menczer F (2011b) Political polarization on twitter In: Proc. 5th Intl. AAAI Conf. on Weblogs and Social Media (ICWSM).

[CR13] Dal Maso C, Pompa G, Puliga M, Riotta G, Chessa A (2014). Voting behavior, coalitions and government strength through a complex network analysis. PLoS ONE.

[CR14] Fortunato S (2010). Community detection in graphs. Phys Reports.

[CR15] Girvan M, Newman MEJ (2002). Community structure in social and biological networks. Proc Nat Acad Sci.

[CR16] Harenberg S, Bello G, Gjeltema L, Ranshous S, Harlalka J, Seay R, Padmanabhan K, Samatova N (2014). Community detection in large-scale networks: a survey and empirical evaluation. Wiley Interdiscip Rev Comput Stat.

[CR17] Hix S, Noury A, Roland G (2009). Voting patterns and alliance formation in the European Parliament. Philos Trans R Soc Lond B Biol Sci.

[CR18] Hric D, Darst RK, Fortunato S (2014). Community detection in networks: Structural communities versus ground truth. Phys Rev E.

[CR19] Kenett DY, Morstatter F, Stanley HE, Liu H (2014). Discovering social events through online attention. PLoS ONE.

[CR20] Kralj Novak P, Smailović J, Sluban B, Mozetič I (2015). Sentiment of emojis. PLoS ONE.

[CR21] Kumar, S, Liu H, Mehta S, Subramaniam LV (2015) Exploring a scalable solution to identifying events in noisy twitter streams In: Proc. IEEE/ACM Intl. Conf. on Advances in Social Network Analysis and Mining (ASONAM), 496–499.

[CR22] Kwak H, Lee C, Park H, Moon S (2010). What is Twitter, a social network or a news media?. Proc. 19th Intl. Conf. on World Wide Web (WWW).

[CR23] Larsson AO (2014). The EU Parliament on Twitter—assessing the permanent online practices of parliamentarians. J Inform Technol Polit.

[CR24] Lazer D (2011). Networks in political science: Back to the future. PS: Pol Sci Polit.

[CR25] Lužar B, Levnajic Z, Povh J, Perc M (2014). Community structure and the evolution of interdisciplinarity in slovenia’s scientific collaboration network. PLoS ONE.

[CR26] Morstatter, F, Pfeffer J, Liu H, Carley KM (2013) Is the sample good enough? comparing data from twitter’s streaming api with twitter’s firehose In: Proc. 7th Intl. AAAI Conf. on Weblogs and Social Media (ICWSM), 400–408.

[CR27] Newman MEJ, Girvan M (2004). Finding and evaluating community structure in networks. Phys Rev E.

[CR28] Newman MEJ (2006). Modularity and community structure in networks. Proc Nat Acad Sci.

[CR29] Peixoto TP (2014). Hierarchical block structures and high-resolution model selection in large networks. Phys Rev X.

[CR30] Perc M (2010). Growth and structure of slovenia’s scientific collaboration network. J Infometrics.

[CR31] Porter MA, Mucha PJ, Newman MEJ, Warmbrand CM (2005). A network analysis of committees in the U.S, House of Representatives. Proc Nat Acad Sci.

[CR32] Ranco G, Aleksovski A, Caldarelli G, Grčar M, Mozetič I (2015). The effects of Twitter sentiment on stock price returns. PLoS ONE.

[CR33] Sampson, J, Morstatter F, Maciejewski R, Liu H (2015) Surpassing the limit: Keyword clustering to improve twitter sample coverage In: Proc. 26th ACM Conf. Hypertext and Social Media (Hypertext), 237–245.

[CR34] Sluban B, Smailović J, Battiston S, Mozetič I (2015). Sentiment leaning of influential communities in social networks. Comput Soc Netw.

[CR35] Smailović, J, Kranjc J, Grčar M, žnidaršič M, Mozetič I (2015) Monitoring the Twitter sentiment during the Bulgarian elections In: Proc. IEEE Intl. Conf. on Data Science and Advanced Analytics (DSAA), 1–10, doi:10.1109/DSAA.2015.7344886.

[CR36] Suh, B, Hong L, Pirolli P, Chi EH (2010) Want to be retweeted? large scale analytics on factors impacting retweet in twitter network In: Proc. 2nd IEEE Intl. Conf. on Social Computing (SocialCom), 177–184.

[CR37] Van Rijsbergen CJ (1979). Information Retrieval.

[CR38] Waugh, AS, Pei L, Fowler JH, Mucha PJ, Porter MA (2009) Party Polarization in Congress: A Network Science Approach. 0907.3509.

[CR39] Weng J, Lim EP, Jiang J, He Q (2010). Twitterrank: Finding topic-sensitive influential twitterers. Proc. 3rd ACM Int. Conf. on Web Search and Data Mining (WSDM).

[CR40] Yang J, Leskovec J (2015). Defining and evaluating network communities based on ground-truth. Knowl Inform Syst.

[CR41] Zhang Y, Friend AJ, Traud AL, Porter MA, Fowler JH, Mucha PJ (2008). Community structure in congressional cosponsorship networks. Physica A: Stat Mech Appl.

[CR42] Zollo F, Kralj Novak P, Del Vicario M, Bessi A, Mozetič I, Scala A, Caldarelli G, Quattrociocchi W (2015). Emotional dynamics in the age of misinformation. PLoS ONE.

